# Reliability of the Frailty Index Among Community-Dwelling Older Adults

**DOI:** 10.1093/gerona/glad227

**Published:** 2023-09-20

**Authors:** Erwin Stolz, Hannes Mayerl, Judith Godin, Emiel O Hoogendijk, Olga Theou, Wolfgang Freidl, Kenneth Rockwood

**Affiliations:** Institute of Social Medicine and Epidemiology, Medical University of Graz, Graz, Austria; Institute of Social Medicine and Epidemiology, Medical University of Graz, Graz, Austria; Geriatric Medicine, Dalhousie University and Nova Scotia Health, Halifax, Nova Scotia, Canada; Department of Epidemiology & Data Science, Amsterdam Public Health Research Institute, Amsterdam UMC-Location VU University Medical Center, Amsterdam, The Netherlands; School of Physiotherapy, Geriatric Medicine, Dalhousie University, Halifax, Nova Scotia, Canada; Institute of Social Medicine and Epidemiology, Medical University of Graz, Graz, Austria; Geriatric Medicine, Department of Medicine, Dalhousie University and Nova Scotia Health, Halifax, Nova Scotia, Canada; (Medical Sciences Section)

**Keywords:** Frail, Internal consistency, Longitudinal, Measurement error, Psychometric, Reliability

## Abstract

**Background:**

Consistent and reproducible estimates of the underlying true level of frailty are essential for risk stratification and monitoring of health changes. The purpose of this study is to examine the reliability of the frailty index (FI).

**Methods:**

A total of 426 community-dwelling older adults from the FRequent health Assessment In Later life (FRAIL70+) study in Austria were interviewed biweekly up to 7 times. Two versions of the FI, one with 49 deficits (baseline), and another with 44 (follow-up) were created. Internal consistency was assessed using confirmatory factor analysis and coefficient omega. Test–retest reliability was assessed with Pearson correlation coefficients and the intraclass correlation coefficient. Measurement error was assessed with the standard error of measurement, limits of agreement, and smallest detectable change.

**Results:**

Participants (64.6% women) were on average 77.2 (±5.4) years old with mean FI_49_ at a baseline of 0.19 (±0.14). Internal consistency (coefficient omega) was 0.81. Correlations between biweekly FI_44_ assessments ranged between 0.86 and 0.94 and reliability (intraclass correlation coefficient) was 0.88. The standard error of measurement was 0.05, and the smallest detectable change and upper limits of agreement were 0.13; the latter is larger than previously reported minimal clinically meaningful changes.

**Conclusions:**

Both internal consistency and reliability of the FI were good, that is, the FI differentiates well between community-dwelling older adults, which is an important requirement for risk stratification for both group-level oriented research and patient-level clinical purposes. Measurement error, however, was large, suggesting that individual health deteriorations or improvements, cannot be reliably detected for FI changes smaller than 0.13.

Frailty describes a state of increased vulnerability to stressors resulting from a cumulative decline in multiple physiological systems among older adults (1). Against the ­background of population aging and increased frailty prevalence in more recent birth cohorts (2,3), the importance of frailty for both public health and clinical practice (4) is expected to increase in the coming years. The frailty index (FI) (5), 1 of the 2 dominant conceptualizations of frailty, is based on the accumulation of a large number of age-related health deficits and ­consistently predicts negative health outcomes such as mortality among older adults (6). FIs based on routine administrative and health record data have been developed in recent years as low-cost and wide-coverage tools to screen for frailty in order to identify those older adults with the highest risk for adverse outcomes (7–11). In addition to risk stratification based on one-time assessments, the FI is also discussed for monitoring health changes in older adults (12–18).

Both risk stratification based on single assessments as well as the evaluation of health changes requires that the degree of frailty in an older person—a latent quality difficult to observe directly—is measured reliably. Reliability can be defined as the extent to which an instrument yields consistent and reproducible estimates of the underlying true score^(p135)^ (19). Multiple systematic reviews (20–23) note that, compared to construct and criterion validity, the reliability of frailty tools has received fairly little attention. However, it is only when we are sure that an instrument measures something in the same way every time we deploy it (=reliability), that we can truly ascertain that it is measuring the right thing (=validity) (24). The COSMIN consensus (25) holds that the domain of reliability consists of 3 different measurement properties: (1) internal consistency, (2) reliability, and (3) measurement error. (1) Internal consistency refers to the degree to which multiple indicators share a common variance due to the underlying construct of frailty, assessed by coefficient alpha or omega (26). (2) Test–retest reliability is the extent to which the relative position of an individual is consistent across multiple time points (24), expressed for example with Pearson’s correlation coefficient or the intraclass correlation coefficient (ICC), and is relevant for discrimination between individuals (27), that is, when the FI is used as a tool for risk stratification. (3) Measurement error, finally, is relevant for frailty monitoring, that is, to differentiate “real” frailty changes from error or “noise,” and can be assessed with the standard error of measurement (SEM) (27). To date, only 2 studies (28,29) provide estimates of the reliability of the standard clinical FI (30). Based on a large cross-national sample of community-dwelling older adults and confirmatory factor analysis (CFA), Mayerl and colleagues (28) reported internal consistency (omega) of 0.89–0.93. Based on 80 stable hospital patients over 3 months, Feenstra et al. (29) reported a test–retest reliability (ICC) of 0.84 and a measurement error (SEM) of 0.06. Although these first studies suggest the FI to be reliable, more evidence is needed against the background of the current and intended future use of the FI in both research and clinical practice.

Here, we use intensive longitudinal data from a nationwide sample of older adults in Austria to provide new evidence on internal consistency, test–retest reliability, and measurement error of the FI among community-dwelling older adults. In this way, we assess the FI’s psychometric properties for risk stratification and monitoring in the context of both group-level research questions and individual-level clinical purposes.

## Method

### Data

Longitudinal data came from the FRequent health Assessment In Later life (FRAIL70+) study. At the behest of the first author, a professional survey agency collected information on health deficits among a nationwide sample of community-dwelling older adults aged 70 years and above in Austria. In total, 971 older adults were contacted based on previous participation in population-representative studies, of which 426 individuals agreed to participate (response rate = 44%; Supplementary Methods 1). Before participation, interviewers described the topic, length, and required information of the study, ensured anonymity of all personal data, and obtained written consent for participation. Between September 2021 and January 2022, participants were interviewed every 2 weeks (mean duration between interviews = 14.7 ± 2.3 days) up to 7 times (mean number of interviews per person = 6.8 ± 0.7), resulting in a total number of 2 892 repeated interviews over a mean period of 84.2 ± 17.0 days (Supplementary Figure 1). The first interview was always an in-person interview conducted in the older adult’s home and included physical performance tests. Six shorter follow-up interviews were conducted via telephone, except for a subsample of 40 older adults, with whom all interviews were conducted in person to obtain repeated physical performance measures and to compare survey modes. This study was approved by the Ethics Committee of the Medical University of Graz (EK-number: 33-243 ex 20/21).

### Variables

Using baseline data, a frailty index (FI_49_) was calculated from 49 health deficits including self-reported information as well as physical and cognitive performance tests following standard protocol (30). This FI_49_ was used to assess internal consistency. Furthermore, a highly similar second FI_44_ based on the subset of those 44 health deficits that were measured repeatedly was created to assess test–retest reliability and measurement error. For both FIs, the selected health deficits reflected multiple physiological systems, and included chronic diseases, limitations in basic and instrumental activities of daily living (ADLs, IADLs), mobility restrictions, somatic symptoms, depressed affect, sensory impairments, physical inactivity, self-rated health, and memory problems (Supplementary Table 1). Self-reported health deficits generally referred to problems or difficulties during the last 2 weeks. All health deficits had less than 2% missing values. The FI score was calculated for all participants by dividing the sum of the health deficit score by the total number of health deficits measured, for example, 10/44 = 0.23. A common cut-off value to differentiate between nonfrail and frail older adults is 0.20 (30).

Sociodemographic variables included sex (male/female), chronological age (years), and level of completed education (low = compulsory education, medium = vocational training, and high = high school or higher). Time since baseline was measured in days. As negative health outcomes, we included 1-year mortality, which was ascertained by proxy interviews or contacting the local municipality. Information on vital status 1 year after participation was 99.5% complete.

### Statistical Analysis

First, we calculated and plotted descriptive statistics for the baseline FI_49_ and the longitudinal FI_44_. Second, we assessed internal consistency. Internal consistency only applies as a measure of reliability, if the multi-item construct under question follows a reflective measurement model, which is linked to criteria (31) such as the direction of causality between construct and indicators, and the interchangeability of and covariation between indicators. In Supplementary Table 2, we outline why we consider the FI to follow a reflective rather than a formative model. Next, as detailed in Supplementary Methods 2, we used polychoric correlations and CFA to test the unidimensionality of the FI prior to calculating internal consistency (coefficient omega). Here, we followed the quality criterion that internal consistency should be greater than 0.80 for population-level research aiming at group comparisons, and greater than 0.90 when individual-level decisions are to be made based on the instrument^(p265)^ (32). Third, we assessed test–retest reliability and measurement error based on the repeated measurements 14-days apart (33,34), a period in which we would not expect substantive frailty changes among community-dwelling older adults; at the same time, memory and learning effects should be limited. For test–retest reliability, as detailed in Supplementary Methods 3, we calculated Pearson correlation coefficients and ICC, and for measurement error, we calculated SEM, limits of agreement (LOA), and smallest detectable change (SDC), all based on the 7 repeated FI_44_ assessments. Here, we followed the quality criterion of an ICC of 0.75–0.90 indicating good reliability, with values above 0.90 being considered excellent (35). For measurement error, clinically meaningful changes (CMC)—that is, differences in continuous measures large enough to be considered important, for example, by clinicians or older adults themselves—should be smaller than the SDC and lie outside the LOA (34). Previous work has suggested CMCs for the FI among community-dwelling older adults of 0.06/0.08 (36) and 0.04/0.06 (37).

All data preparation, calculations, and statistical tests were done with R (v4.3.0), which are documented in the R-Markdown code file available online: https://osf.io/qvek2/.

## Results

### Sample Characteristics and Descriptive Statistics

**Figure 1.**
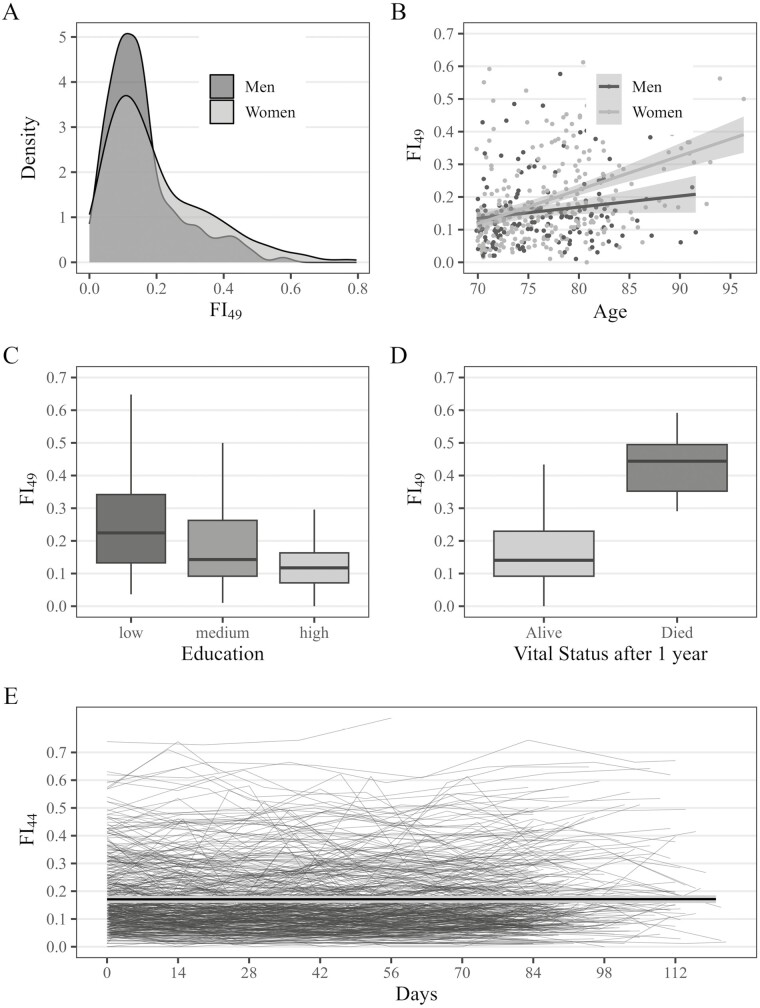
Descriptive statistics of the baseline frailty index (FI_49_) and the longitudinal frailty index (FI_44_). FI_49_ = frailty index at baseline based on 49 health deficits; FI_44_ = longitudinal frailty index based on the same 44 health deficits in all 7 repeated assessment. Estimated overall trajectory in plot E is the fitted mean trajectory based on a linear mixed model, and the light gray shaded area indicates 95% confidence intervals.

Of 426 participants at baseline, 64.6% were women, with a mean age of 77.3 (±5.4, range = 70–96) years. Low education was reported by 19.3%, medium by 54.2%, and high by 26.5%. The mean (*SD*) and median interquartile range (IQR) of the FI_49_ were 0.19 (±0.14) and 0.14 (±0.16). The empirical submaximum (99^th^ percentile) was 0.63. The prevalence of specific health deficits at baseline is shown in Supplementary Table 1. The FI_49_ exhibited a right-skewed distribution, with higher values among women than men (Figure 1A), and a positive relationship with age, with a steeper slope for women than men (Figure 1B). Older adults with a low level of education had higher mean FI_49_ values at baseline (0.25 ± 0.16) compared to those who had completed vocational training (0.19 ± 0.14), which again were frailer compared to those who had completed upper secondary or higher education (0.13 ± 0.08; Figure 1C). Participants who died during 1-year follow-up (*n* = 11, 2.6%) had a substantively higher median FI_49_ (0.47 ± 0.20) compared to those who survived (0.18 ± 0.14; Figure 1D). Based on logistic regression analysis adjusted for age, the odds of death were 11% higher (OR = 1.11, 95% CI: = 1.07–1.17) per 0.01 FI points.

**Figure 2.**
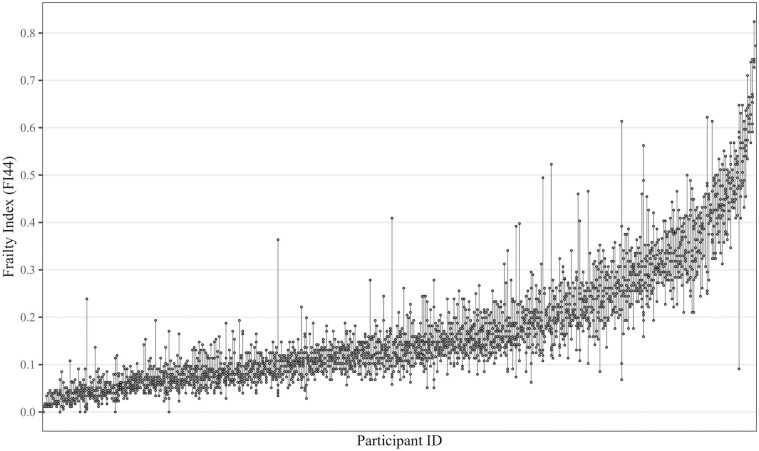
Repeated frailty index (FI_44_) assessments by participant. FI_44_ = longitudinal frailty index based on the same 44 health deficits in all 7 assessments. Points show repeated FI_44_ assessments for each person, each line represents 1 participant. Participants are ordered according to their mean FI_44_.

The correlation between the FI_49_ and FI_44_ at baseline was 0.99 (95% CI: = 0.99, 0.99). Descriptive statistics of the longitudinal FI_44_ for each assessment (Table 1) showed little change in the average frailty level across biweekly assessments. We also found no evidence of a linear change in the overall level of frailty across the 3 months (Figure 1E). There were, however, considerable within-person instabilities or fluctuations visible, particularly in the higher FI regions (Figure 2) readily seen when repeated FI_44_ assessments (points) for each person (lines) were ordered by their mean FI_44_ level. Finally, we found that both mean FI_44_ change and individual FI_44_ fluctuations were similar in both interview modes (Supplementary Figure 2).

**Table 1.** Descriptive Statistics of the Frailty Index (FI_44_) by Measurement Occasion

Assessment #	Sample size	Mean (*SD*)	Median (IQR)	99%tile
1	426	0.18 (0.13)	0.14 (0.15)	0.60
2	418	0.17 (0.13)	0.12 (0.15)	0.64
3	419	0.16 (0.13)	0.12 (0.13)	0.62
4	410	0.17 (0.13)	0.12 (0.15)	0.59
5	406	0.16 (0.12)	0.12 (0.13)	0.57
6	407	0.17 (0.13)	0.12 (0.15)	0.56
7	406	0.17 (0.13)	0.14 (0.15)	0.60

*Notes*: IQR = interquartile range; *SD* = standard deviation; 99%tile = 99th percentile, that is, the empirical submaximum.

Unweighted data, FI based on 44 health items.

### Internal Consistency

The mean overall polychoric correlation among health deficits was 0.29 (±0.18), which is adequate (38) for a broad construct such as the FI (Supplementary Figure 3). The mean polyserial correlation between FI_49_ and its health deficits was 0.50 (±0.17), which again meets the criteria for scale construction^(p93)^ (33). The highest correlation coefficients were observed for poor self-reported health as well as ADL, IADL, and mobility impairments including slow gait speed (range = 0.60–0.70), whereas lower associations were found for chronic diseases (range = 0.20–0.30) (Supplementary Table 3).

Next, we tested whether the FI can be assumed a unidimensional measure. Comparison of a unidimensional single-factor model with a multidimensional correlated factor/first-order model of 3 separated domains (physical, cognitive, and mental health) without a superstructure, and a bifactor model that retains a general factor of frailty as well as remaining subdomain variance showed (Supplementary Table 3) the bifactor model to fit best (χ² = 1 409, *df* = 1 121, *p* < .001, CFI = 0.97, TLI = 0.97, RMSEA = 0.02, SRMR = 0.107). In addition, the factor loadings between the unidimensional model and the general factor of the bifactor model were closely correlated (*r* = 0.96), and 87% of the reliable variance (omega of the general factor in the bifactor model divided by omega of the 1-factor model, 0.81/0.93 = 87%) in the health deficits was due to the general factor, which suggests that the FI is unidimensional enough for practical purposes. Internal consistency reliability for the general factor depicting overall frailty as measured by coefficient omega (26) was 0.81, which is good.

More detailed results from the bifactor CFA model (Supplementary Table 4) also show how well specific health deficits reflected the overall frailty level. The highest factor loadings showed for ADLs (eg, using the toilet = 0.88), IADLs (eg, preparing a warm meal = 0.87), self-rated health (0.83), and polypharmacy (0.83). Loadings that were more moderate showed for bedrest (0.69), tiredness (0.64), physical inactivity (0.62), poor appetite (0.54), and attention (0.47) and memory (0.41) problems. Finally, chronic diseases had—except for arthritis (0.44) and dementia (0.47)—notably lower loadings between 0.20 and 0.30, the lowest being cancer (0.14).

### Reliability

**Figure 3.**
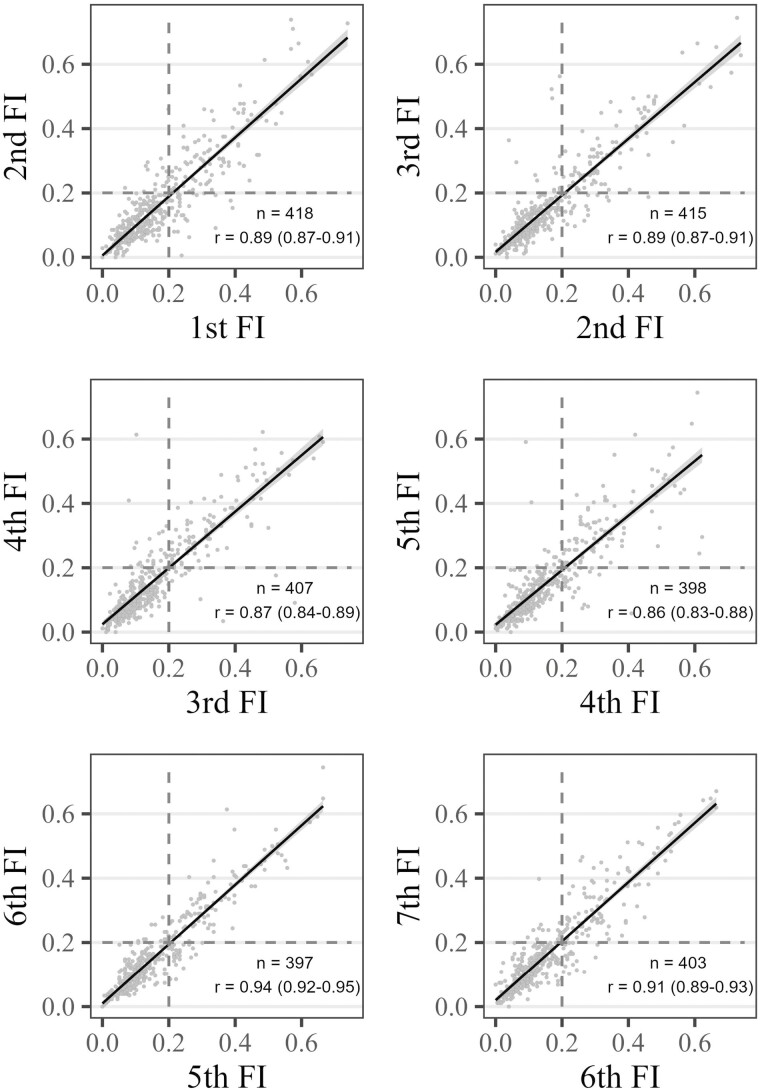
Correlations between subsequent frailty index (FI_44_) measurements. FI = frailty index based on 44 health deficits, *n* = sample size in paired assessments, *r* = Pearson’s correlation coefficient, values in parentheses are 95% confidence intervals. Dashed lines indicate the cut-off to differentiate between nonfrail and frail older adults.

The Pearson correlation coefficients between adjacent FI assessments (Figure 3) showed a strong association, ranging between 0.86 and 0.89 among the first 4 assessments, and reaching 0.94 and 0.91 between the last 3 assessments. Nonetheless, using 0.20 as a cut-off for frailty (dashed lines), showed that 11%–18% of participants would be classified incoherently—that is, one time as frail and the other time as nonfrail—across assessments only 14 days apart.

Results from the linear mixed regression model showed that the largest part of the total FI variance was between person differences ($\sigma_{i}^{2}\;$= 0.125), followed by the error variance $(\sigma_{residual}^{2}$ = 0.05), whereas there was no systematic variation across waves ($\sigma_{j}^{2}$ = 0.004). The ICC was 0.88 (95% CI: = 0.86–0.90), which can be considered very good.

### Measurement Error

**Figure 4.**
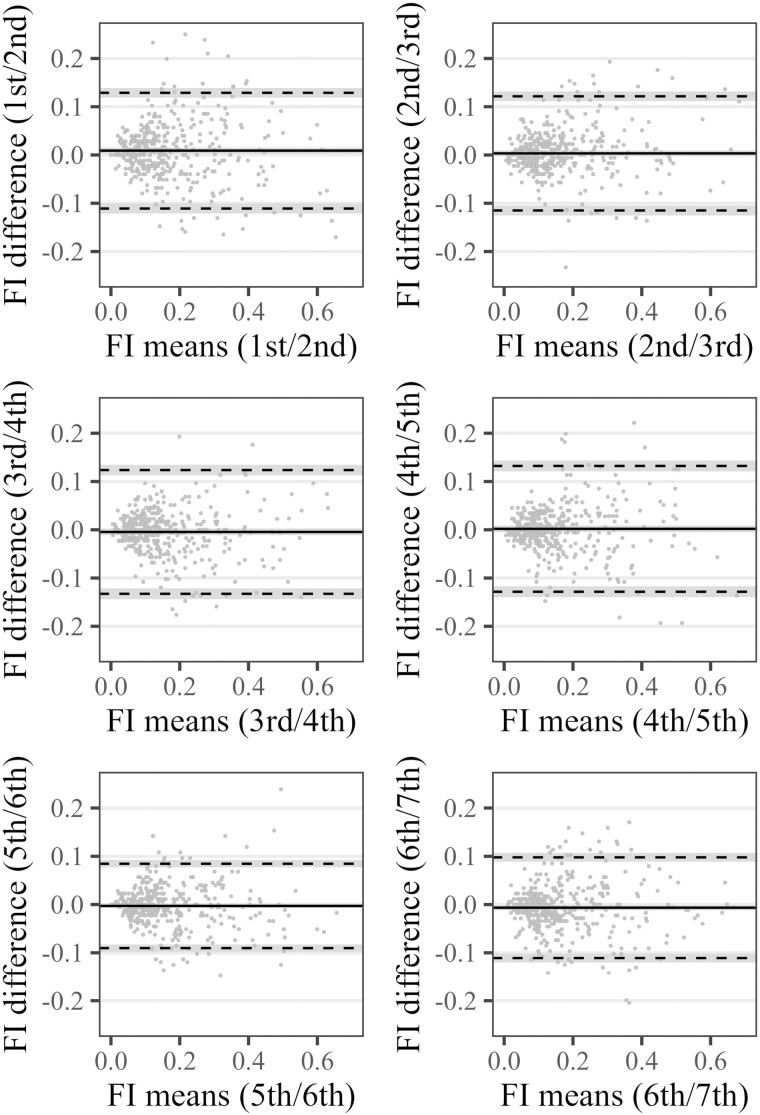
Limits of agreement between subsequent frailty index (FI_44_) measurements (Bland–Altman plots). FI = frailty index based on 44 health deficits. Solid lines shows the extent of systematic bias between paired assessments, dashed lines indicate upper and lower limit of agreement which contains 95% of the paired FI differences, shaded area indicates 95% confidence intervals.

The SEM was 0.046 (95% CI: = 0.045–0.047) and the SDC was 0.127 (95% CI: = 0.125, 0.130). The latter value means that a FI change of at least 0.13 needs to occur to be (95%) confident, that this change is real and not just due to the measurement error of the instrument. These results were also reflected in the Bland–Altman plots (Figure 4) between adjacent FI assessments. There was no indication of systematic bias, and the larger of the two LOA, which together encompass 95% of the paired observations, ranged between 0.09 and 0.13 across waves. Anchor-/distribution-based CMCs provided in the literature (36,37) for community-dwelling older adults—0.06/0.08, respectively, 0.04/0.06—were clearly smaller than the SDC, and lay within the LOA in our study, which means that such FI changes (0.06 for example equates to 2.6 deficits) cannot be reliably differentiated from measurement error. Only changes larger than 0.13 (or 5.7 deficits) in individuals can be confidently interpreted as real changes.

Finally, since short-term fluctuations across biweekly FI assessments seemed more pronounced in frail compared to nonfrail older adults (Figure 2), we also calculated ICC and SEM according to baseline FI_44_ differences (<0.20/≥0.20). The ICC was smaller for both subgroups compared to the total sample—because the between-person variance became smaller due to the partitioning—but the effect was stronger among nonfrail (0.65, 95% CI: = 0.60, 0.69) compared to frail (0.80, 95% CI: = 0.75, 0.84) participants. In contrast, the SEM was smaller among those who were nonfrail at baseline (SEM = 0.037, 95% CI: = 0.036, 0.038) compared to frail participants (SEM = 0.062, 95% CI: = 0.058, 0.065), as were the SDCs: 0.102 (95% CI: = 0.099, 0.105) versus 0.171 (95% CI: = 0.162, 0.179).

## Discussion

In this study, we found internal consistency and test–retest reliability of the FI to be good, respectively, very good. This means that the standard clinical FI under study was able to differentiate well between groups and individuals of community-dwelling older adults, which is an important requirement for risk stratification. The measurement error, however, was relatively large, so only changes above 0.13 in the FI instrument can be safely interpreted as real improvements or deteriorations among individuals. At higher degrees of frailty, differentiating between older adults was easier due to the larger differences between them, while evaluating their health changes was more difficult, as larger health changes were necessary to differentiate genuine health deterioration or improvement from the noise given their high(er) short-term within-person variability. It should be intuitive that what is a meaningful change needs to be standardized in relation to where it is on the scale, reflecting that as with many age-related attributes, variability increases with the degree of frailty.

The first measure of reliability we assessed was internal consistency, which assumes a reflective measurement model (31), which among other factors, depends on the exchangeability of indicators. In contrast to other frailty instruments, particularly phenotypic frailty (39) which is defined by 5 specific indicators (weight loss, exhaustion, weakness, slow gait, and low physical activity), the health deficits of the FI can be seen as manifestations rather than defining characteristics, and are hence in principle exchangeable (30). Another indicator of a reflective measurement model is positive correlations among indicators and between indicators and the overall scale, for which we both found evidence. Using CFA, we tested the unidimensionality of the FI before assessing internal consistency. We found the FI to be essentially unidimensional, although future studies should psychometrically vet the choice of health deficits for the construction of clinical FIs more thoroughly, for example using item response models, to ensure that the best set of indicators for overall frailty are put to use in both research and practice (40). Here, we found that health deficits loaded differentially on the single underlying factor frailty: poor self-reported health and restrictions in ADLs and IADLs as well as mobility reflected overall frailty best, a finding that is also supported by network analyses of the FI, where these deficits are found to integrate many systems (41,42). With the exception of dementia and arthritis, many chronic diseases, on the other hand, contributed notably less to overall frailty, particularly cancer.

Despite generally limited evidence on the reliability of frailty instruments (20–23), a few studies offer interesting points for comparison. First, our findings on internal consistency are highly similar to those from Mayerl et al. (28) with regard to the 1-factor model. In the final bifactor model where we adjusted for the multidomain nature of the FI, we still found a good level of internal consistency (0.81). Among other frailty instruments, internal consistency tends to be smaller, for example, 0.62 in the Edmonton Frail Scale (43) or 0.66–0.80 in the Tilburg Frailty Indicator (44). This is likely due to the often fewer indicators considered in these tools, as coefficient alpha and omega are not only a function of the interrelatedness of the indicators, but also their number. Indeed, Nguyen et al. (45) showed in a simulation study comparing various FI configurations, that the reliability of the FI is associated with the number of health deficits considered, ranging from ICC = 0.19 with just 5 health deficits up to ICC = 0.84 with 45.

Test–retest reliability of the FI over multiple 14-day periods was ICC = 0.88, which is slightly above the results reported for stable hospital patients over 3 months (ICC = 0.84/0.85) (29). Our estimate also compares favorably with the range of test–retest ICCs reported for other frailty instruments, for example, 0.65/0.77 for phenotypic frailty over 3 months (29), 0.71 for the FRAIL scale over 7–15 days (46), and 0.88 for the Tilburg Frailty Indicator over 10–25 days (47). In sum, test–retest reliability, as well as internal consistency of the FI were good, and hence the FI can be considered a high-quality instrument for risk stratification among older adults. The good reliability of the FI means that it lends itself well for the assessment of group-level differences in research, for example, to identify risk factors or population-health management, for example, to implement prevention programs to halt or decrease health deterioration among particularly vulnerable older adults (4). Given the high level of test–retest reliability, the FI likely can also be employed as a tool to inform individual-level clinical decision-making (48), that is, tailoring interventions to the frailty level, for example by avoiding aggressive treatments among the most vulnerable patients, and by providing goal-oriented and coordinated care.

For measurement error, we found a SEM of 0.05, and upper LOA and SDC values of 0.13 for the FI, which correspond closely to the results of Feenstra et al. (29) The evaluation of the latter values depends on the magnitude of CMCs for the FI (34). For community-dwelling older adults, CMCs of 0.06/0.08 (36) and 0.04/0.06 (37) have been suggested. Since these CMCs fall within the LOA respectively and are clearly smaller than the SDC in our study, as well as in the work of Feenstra et al. (29), the measurement error of the FI must be considered substantial. The FI as a broad summary measure of an older person’s overall health status (5) seems not well suited for monitoring such health changes of 0.04–0.08 in single individuals accurately, that is, health deteriorations of about 2–4 deficits (in our FI_44_) would not be enough to be clearly differentiated from measurement error or the noise of the short-term fluctuations we found. More conservatively, the SDC in the FI that signifies a real deterioration or improvement in the FI in a presenting individual would need to amount to 0.13, or about 6 health deficits. Among frail older adults (FI > 0.20), it is even more difficult to measure and interpret individual-level health changes reliably. This considerably large measurement error of the FI, however, is unlikely to affect research interested in risk factors for FI trajectories such as sex, socioeconomic status, or BMI categories (49) as the SEM for group differences in FI trajectories will be much smaller than for single individuals. This applies even if reversible fluctuations are more prevalent in some groups than others (50). The relatively large measurement error, may, however, limit the FI’s potential for accurate individual-level monitoring, for example, based on electronic routine health data (13). It might be helpful to view any single FI score from an individual as just 1 data point in a long string of unmeasured FIs that may fluctuate considerably around the one realized measurement. To reduce the measurement error of the FI, (1) more health deficits could be used, (2) more test-based indicators, which come with less measurement error than self-reports, could be incorporated, and (3) information loss could be reduced by avoiding dichotomization of health deficits if possible (51). Furthermore, future research should systematically assess which health deficits are fueling the observed short-term instability of the FI, and weigh their added value for the FI, for example by assessing the loadings of individual health deficits on the FI, against the instability associated with such indicators. Cooper et al. (48), for example, decided to remove patient-reported low mood in their clinical implementation of the FI due to its short-term variability.

However, the aforementioned within-person FI fluctuations, which have been described earlier (52) and which appear related to the FI level, could also be more than just noise (50). Not only could these FI fluctuations reflect chains of discrete health transitions over weeks and months, for example, from high functioning to acute illness or injury, followed by hospitalization, and recovery (53), but they may also be driven by age-related fluctuations inherent in disability (54), somatic symptoms (55), or cognition (56), which tend to be also associated with negative health outcomes. Hence, future studies should not only investigate how these instabilities come about and how to limit their influence but also to find out whether these seemingly stochastic fluctuations could be a relevant characteristic of system failure on their own.

The current study has several strengths. We used a nationwide cohort study of community-dwelling older adults where the FI was assessed multiple times over 2-week periods, and the sample size was large for a reliability study. Also, this is the first time that information on all 3 properties of reliability (25) (internal consistency, reliability, and measurement error) of the FI was reported within a single study. Noteworthy limitations include that although nationwide data were collected, there were selection effects insofar as women, higher educated, and younger persons were somewhat overrepresented in the FRAIL70+ sample. Such selection effects, however, are common in health and aging survey studies, and we consider it unlikely that these affected the estimation of the reliability measures substantively. Furthermore, the longitudinal FI_44_ consisted only of self-reported health problems except for 3 cognitive tests, which could influence the extent of short-term FI fluctuations, and in turn, may have affected our reliability estimates. Given the smaller measurement error of physical performance tests compared to self-reports, our results can therefore be interpreted as a conservative, ­lower-end estimate of the FI’s reliability.

## Conclusion

Both internal consistency as well as test–retest reliability were good, that is, the FI differentiates well between community-dwelling older adults, which is an important requirement for risk stratification for both research and clinical purposes. Measurement error was considerable though, which means that smaller FI changes among individuals cannot be identified reliably. Furthermore, we uncovered considerable reversible short-term fluctuations in the FI which merit further study.

## Supplementary Material

glad227_suppl_Supplementary_Material

## Data Availability

All data and the R-code to reproduce all analyses and results are available online (https://osf.io/qvek2/).

## References

[1] Clegg A , Young J, Iliffe S, Rikkert MO, Rockwood K. Frailty in elderly people. Lancet. 2013;381(9868):752–762. 10.1016/S0140-6736(12)62167-9 23395245 PMC4098658

[2] Yu R , Wong M, Chong KC, et al. Trajectories of frailty among Chinese older people in Hong Kong between 2001 and 2012: an age-period-cohort analysis. Age Ageing. 2018;47(2):254–261. 10.1093/ageing/afx170 29161361

[3] Hoogendijk EO , Stolz E, Oude Voshaar RC, Deeg DJH, Huisman M, Jeuring HW. Trends in frailty and its association with mortality: results from the longitudinal aging study Amsterdam, 1995–2016. Am J Epidemiol. 2021;190(7):1316–1323. 10.1093/aje/kwab018 33534876 PMC8245891

[4] Hoogendijk EO , Afilalo J, Ensrud KE, Kowal P, Onder G, Fried LP. Frailty: implications for clinical practice and public health. Lancet. 2019;394(10206):1365–1375. 10.1016/S0140-6736(19)31786-6 31609228

[5] Rockwood K , Mitnitski A. Frailty in relation to the accumulation of deficits. J Gerontol A Biol Sci Med Sci. 2007;62(7):722–727. 10.1093/gerona/62.7.722 17634318

[6] Kojima G , Iliffe S, Walters K. Frailty index as a predictor of mortality: a systematic review and meta-analysis. Age Ageing. 2018;47(2):193–200. 10.1093/ageing/afx162 29040347

[7] Clegg A , Bates C, Young J, et al. Development and validation of an electronic frailty index using routine primary care electronic health record data. Age Ageing. 2016;45(3):353–360. 10.1093/ageing/afw039 26944937 PMC4846793

[8] Gilbert T , Neuburger J, Kraindler J, et al. Development and validation of a Hospital Frailty Risk Score focusing on older people in acute care settings using electronic hospital records: an observational study. Lancet. 2018;391(10132):1775–1782. 10.1016/S0140-6736(18)30668-8 29706364 PMC5946808

[9] Pajewski NM , Lenoir K, Wells BJ, Williamson JD, Callahan KE. Frailty screening using the electronic health record within a medicare accountable care organization. J Gerontol A Biol Sci Med Sci. 2019;74(11):1771–1777. 10.1093/gerona/glz017 30668637 PMC6777083

[10] Kim DH , Patorno E, Pawar A, Lee H, Schneeweiss S, Glynn RJ. Measuring frailty in administrative claims data: comparative performance of four claims-based frailty measures in the United States medicare data. J Gerontol A Biol Sci Med Sci. 2020;75:1120–1125. 10.1093/gerona/glz224 31566201 PMC7243587

[11] Mak JKL , Hägg S, Eriksdotter M, et al. Development of an electronic frailty index for hospitalized older adults in Sweden. J Gerontol A Biol Sci Med Sci. 2022;77(11):2311–2319. 10.1093/gerona/glac069 35303746 PMC9678204

[12] Hoogendijk EO , Rockwood K, Theou O, et al. Tracking changes in frailty throughout later life: results from a 17-year longitudinal study in the Netherlands. Age Ageing. 2018;47(5):727–733. 10.1093/ageing/afy081 29788032

[13] Stow D , Matthews FE, Hanratty B. Frailty trajectories to identify end of life: a longitudinal population-based study. BMC Med. 2018;16(1):171. 10.1186/s12916-018-1148-x 30236103 PMC6148780

[14] Thompson MQ , Theou O, Tucker GR, Adams RJ, Visvanathan R. Recurrent measurement of frailty is important for mortality prediction: findings from the North West Adelaide Health Study. J Am Geriatr Soc. 2019;67:2311–2317. 10.1111/jgs.16066 31317527

[15] Stolz E , Hoogendijk EO, Mayerl H, Freidl W. Frailty changes predict mortality in 4 longitudinal studies of aging. J Gerontol A Biol Sci Med Sci. 2020;76:1619–1626. 10.1093/gerona/glaa266 PMC8361367 33103718

[16] Bai G , Szwajda A, Wang Y, et al. Frailty trajectories in three longitudinal studies of aging: is the level or the rate of change more predictive of mortality? Age Ageing. 2021;50:2174–2182. 10.1093/ageing/afab106 34120182 PMC8581383

[17] Shi SM , Olivieri-Mui B, McCarthy EP, Kim DH. Changes in a frailty index and association with mortality. J Am Geriatr Soc. 2021;69(4):1057–1062. 10.1111/jgs.17002 33377190 PMC8071066

[18] Stolz E , Mayerl H, Hoogendijk EO. Frailty in the oldest old: is the current level or the rate of change more predictive of mortality? Age Ageing. 2022;51(2):afac020. 10.1093/ageing/afac020 35165691

[19] Krabbe PFM. The Measurement of Health and Health Status. Concepts, Methods, and Applications from a Multidisciplinary Perspective. London: Elsevier; 2017.

[20] de Vries NM , Staal JB, van Ravensberg CD, Hobbelen JSM, Olde Rikkert MGM, Nijhuis-van der Sanden MWG. Outcome instruments to measure frailty: a systematic review. Ageing Res Rev. 2011;10(1):104–114. 10.1016/j.arr.2010.09.001 20850567

[21] Bouillon K , Kivimaki M, Hamer M, et al. Measures of frailty in population-based studies: an overview. BMC Geriatr. 2013;13:64. 10.1186/1471-2318-13-64 23786540 PMC3710231

[22] Sutton JL , Gould RL, Daley S, et al. Psychometric properties of multicomponent tools designed to assess frailty in older adults: a systematic review. BMC Geriatr. 2016;16:55. 10.1186/s12877-016-0225-2 26927924 PMC4772336

[23] Ambagtsheer RC , Thompson MQ, Archibald MM, Casey MG, Schultz TJ. Diagnostic test accuracy of self-reported screening instruments in identifying frailty in community-dwelling older people: A systematic review. Geriatr Gerontol Int. 2020;20(1):14–24. 10.1111/ggi.13810 31729157

[24] Aldridge VK , Dovey TM, Wade A. Assessing test-retest reliability of psychological measures: Persistent methodological problems. Eur Psychol. 2017;22:207–218. 10.1027/1016-9040/a000298

[25] Mokkink LB , Terwee CB, Patrick DL, et al. The COSMIN study reached international consensus on taxonomy, terminology, and definitions of measurement properties for health-related patient-reported outcomes. J Clin Epidemiol. 2010;63(7):737–745. 10.1016/j.jclinepi.2010.02.006 20494804

[26] Revelle W , Condon DM. Reliability from α to ω: a tutorial. Psychol Assess. 2019;31:1395–1411. 10.1037/pas0000754 31380696

[27] de Vet HCW , Terwee CB, Knol DL, Bouter LM. When to use agreement versus reliability measures. J Clin Epidemiol. 2006;59(10):1033–1039. 10.1016/j.jclinepi.2005.10.015 16980142

[28] Mayerl H , Stolz E, Freidl W. Frailty and depression: reciprocal influences or common causes? Soc Sci Med. 2020;263:113273. 10.1016/j.socscimed.2020.113273 32810695

[29] Feenstra M , Oud FMM, Jansen CJ, Smidt N, van Munster BC, de Rooij SE. Reproducibility and responsiveness of the Frailty Index and Frailty Phenotype in older hospitalized patients. BMC Geriatr. 2021;21(1):499. 10.1186/s12877-021-02444-y 34535074 PMC8447764

[30] Searle SD , Mitnitski A, Gahbauer EA, Gill TM, Rockwood K. A standard procedure for creating a frailty index. BMC Geriatr. 2008;8(1):24. 10.1186/1471-2318-8-24 18826625 PMC2573877

[31] Fleuren BPI , van Amelsvoort LGPM, Zijlstra FRH, de Grip A, Kant I. Handling the reflective-formative measurement conundrum: a practical illustration based on sustainable employability. J Clin Epidemiol. 2018;103:71–81. 10.1016/j.jclinepi.2018.07.007 30031210

[32] Nunnally JC , Bernstein IH. Psychometric Theory. 3rd ed. McGraw-Hill; 1994. Accessed February 16, 2023. http://catdir.loc.gov/catdir/toc/mh022/93022756.html

[33] Streiner DL , Norman GR, Cairney J. Health Measurement Scales: A Practical Guide to Their Development and Use, 5th Ed. Oxford University Press; 2015:xiii, 399. 10.1093/med/9780199685219.001.0001

[34] Terwee CB , Bot SDM, de Boer MR, et al. Quality criteria were proposed for measurement properties of health status questionnaires. J Clin Epidemiol. 2007;60(1):34–42. 10.1016/j.jclinepi.2006.03.012 17161752

[35] Koo TK , Li MY. A guideline of selecting and reporting intraclass correlation coefficients for reliability research. J Chiropr Med. 2016;15(2):155–163. 10.1016/j.jcm.2016.02.012 27330520 PMC4913118

[36] Jang IY , Jung HW, Lee HY, Park H, Lee E, Kim DH. Evaluation of clinically meaningful changes in measures of frailty. J Gerontol A Biol Sci Med Sci. 2020;75(6):1143–1147. 10.1093/gerona/glaa003 32145016 PMC7243580

[37] Thompson MQ , Theou O, Ratcliffe J, et al. Frailty state utility and minimally important difference: findings from the North West Adelaide Health Study. Age Ageing. 2021;50(2):565–569. 10.1093/ageing/afaa166 32936870

[38] Clark LA , Watson D. Constructing validity: basic issues in objective scale development. Psychol Assess. 1995;7:309–319. 10.1037/1040-3590.7.3.309

[39] Fried LP , Tangen CM, Walston J, et al.; Cardiovascular Health Study Collaborative Research Group. Frailty in older adults evidence for a phenotype. J Gerontol A Biol Sci Med Sci. 2001;56(3):M146–M156. 10.1093/gerona/56.3.m146 11253156

[40] Mayo NE , Aubertin-Leheudre M, Mate K, et al. Development of a frailty ladder using Rasch analysis: if the shoe fits. Can Geriatr J. 2023;26(1):133–143. 10.5770/cgj.26.601 36865407 PMC9953502

[41] Farrell SG , Mitnitski AB, Theou O, Rockwood K, Rutenberg AD. Probing the network structure of health deficits in human aging. Phys Rev E. 2018;98(3):032302. 10.1103/PhysRevE.98.032302

[42] García-Peña C , Ramírez-Aldana R, Parra-Rodriguez L, Gomez-Verjan JC, Pérez-Zepeda MU, Gutiérrez-Robledo LM. Network analysis of frailty and aging: empirical data from the Mexican Health and Aging Study. Exp Gerontol. 2019;128:110747. 10.1016/j.exger.2019.110747 31665658 PMC7493650

[43] Rolfson DB , Majumdar SR, Tsuyuki RT, Tahir A, Rockwood K. Validity and reliability of the Edmonton Frail Scale. Age Ageing. 2006;35(5):526–529. 10.1093/ageing/afl041 16757522 PMC5955195

[44] Gobbens RJ , Uchmanowicz I. Assessing Frailty with the Tilburg Frailty Indicator (TFI): a review of reliability and validity. Clin Interv Aging. 2021;16:863–875. 10.2147/CIA.S298191 34040363 PMC8140902

[45] Nguyen QD , Moodie EM, Keezer MR, Wolfson C. Clinical correlates and implications of the reliability of the frailty index in the Canadian Longitudinal Study on Aging. J Gerontol A Biol Sci Med Sci. 2021;76(11):e340–e346. 10.1093/gerona/glab161 34097017 PMC8514068

[46] Dong L , Qiao X, Tian X, et al. Cross-cultural adaptation and validation of the FRAIL scale in Chinese community-dwelling older adults. J Am Med Dir Assoc. 2018;19(1):12–17. 10.1016/j.jamda.2017.06.011 28757330

[47] Dong L , Liu N, Tian X, et al. Reliability and validity of the Tilburg Frailty Indicator (TFI) among Chinese community-dwelling older people. Arch Gerontol Geriatr. 2017;73:21–28. 10.1016/j.archger.2017.07.001 28734173

[48] Cooper L , Loewenthal J, Frain LN, et al. From research to bedside: incorporation of a CGA-based frailty index among multiple comanagement services. J Am Geriatr Soc. 2022;70(1):90–98. 10.1111/jgs.17446 34519037 PMC9056009

[49] Welstead M , Jenkins ND, Russ TC, Luciano M, Muniz-Terrera G. A systematic review of frailty trajectories: their shape and influencing factors. Gerontologist. 2021;61(8):e463–e475. 10.1093/geront/gnaa061 32485739 PMC8599181

[50] Stolz E , Mayerl H, Freidl W. Fluctuations in frailty among older adults. Age Ageing. 2019;48(4):547–552. 10.1093/ageing/afz040 31028381

[51] Stubbings G , Rockwood K, Mitnitski A, Rutenberg A. A quantile frailty index without dichotomization. Mech Ageing Dev. 2021;199:111570. 10.1016/j.mad.2021.111570 34517019

[52] Mitnitski A , Song X, Rockwood K. Trajectories of changes over twelve years in the health status of Canadians from late middle age. Exp Gerontol. 2012;47(12):893–899. 10.1016/j.exger.2012.06.015 22790020

[53] Gill TM , Allore HG, Gahbauer EA, Murphy TE. Change in disability after hospitalization or restricted activity in older persons. JAMA. 2010;304(17):1919–1928. 10.1001/jama.2010.1568 21045098 PMC3124926

[54] Stolz E , Gill TM, Mayerl H, Freidl W. Short-term disability fluctuations in late life. J Gerontol B Psychol Sci Soc Sci. 2019;74(8):e135–e140. 10.1093/geronb/gbz089 31298701 PMC6777769

[55] Slavish DC , Taylor DJ, Lichstein KL. Intraindividual variability in sleep and comorbid medical and mental health conditions. Sleep. 2019;42(6):zsz052. 10.1093/sleep/zsz052 30843059 PMC6559172

[56] Salthouse TA , Nesselroade JR, Berish DE. Short-term variability in cognitive performance and the calibration of longitudinal change. J Gerontol B Psychol Sci Soc Sci. 2006;61(3):P144–P151. 10.1093/geronb/61.3.p144 16670183 PMC3838959

