# Supplementary material for: Reliability of the Frailty Index Among Community-Dwelling Older Adults
Source: J Gerontol A Biol Sci Med Sci. 2023 Sep 20;79(2):glad227. doi: 10.1093/gerona/glad227 (PMC10809054; doi:10.1093/gerona/glad227)
Supplement: glad227_suppl_Supplementary_Material [file glad227_suppl_supplementary_material.docx]

**Supplementary Material**

Supplementary Methods 1: Sample selection and comparison with population data

Supplementary Figure 1: Interview schedule

Supplementary Table 1: Health deficits of the FI

Supplementary Table 2: Criteria distinguishing reflective and formative concepts and their application to the FI

Supplementary Methods 2: Calculation of internal consistency reliability

Supplementary Methods 3: Calculation of test-retest reliability and measurement error

Supplementary Figure 2: Descriptive statistics of the longitudinal frailty index (FI_44_) by interview mode

Supplementary Table 3: Model fit comparison (confirmatory factor analysis)

Supplementary Table 4: Bivariate correlations and factor loadings (FI_49_)

Supplementary Figure 3: Polychoric correlation matrix (FI_49_)

Supplementary References

Supplementary Methods 1: Sample selection and comparison with population data

When selected older adults were successfully contacted but did not want to participate in the full study, they were asked to provide at least basic demographic and health-related information, so that we might better understand selection into the sample. In comparison to participants, those who could or would not participate were more likely to be male (42.8% vs. 35.4%; χ²=5.3, df=1, p=0.021), to have had only minimum compulsory schooling (28.1% vs. 19.2%; χ²=20.3, df=2, p<0.001), and to have poorer self-reported health (moderate = 37.5% vs. 28.4%, poor = 11.1% vs. 10.8%; χ²=23.9, df=4, p<0.001), but were of similar age (mean=77.4 vs. 77.3 years; F-statistic=0.19, df=1, p=0.663). In the FRAIL70+ study (n=426), compared to the general population aged 70 years and above in Austria, women (65% instead of 58%) and those who finished upper secondary or tertiary education (27% instead of 14%) were overrepresented. Also, the sample was on average one year younger (mean age=77.3 years) than the overall population (78.3 years).

Supplementary Figure 1: Interview schedule


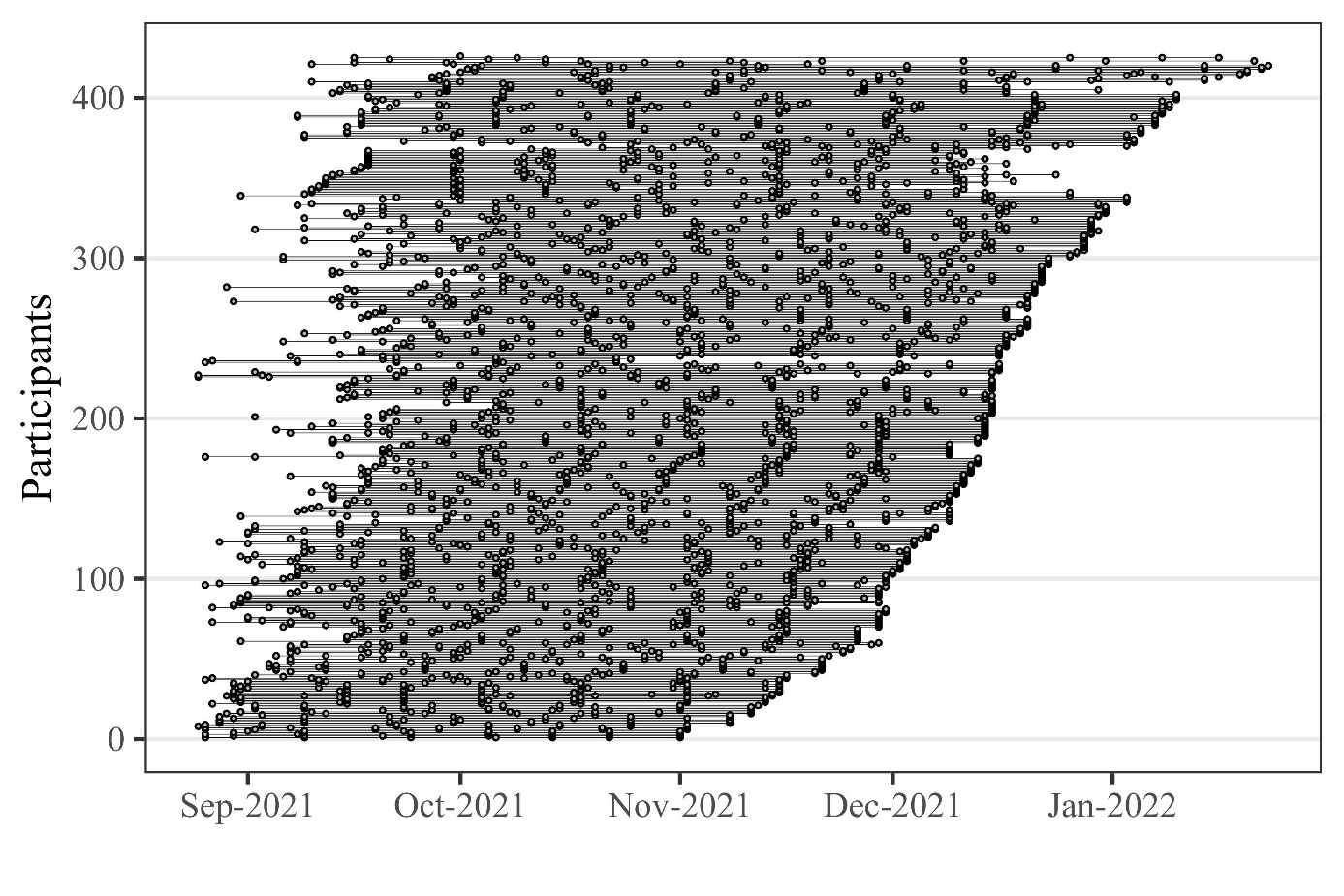


Each grey line shows a participant, each circle a completed interview.

Supplementary Table 1: Health deficits of the FI

| Health deficit | Coding | Prevalence at baseline in % | Missing data at baseline in % |
| --- | --- | --- | --- |
| Self-rated health | Excellent = 0, very good = 0.25, good = 0.50, moderate = 0.75, poor = 1 | 0 = 6.3  0.25 = 18.5  0.50 = 35.9  0.75 = 28.4  1 = 10.8 | - |
| Polypharmacy (measured only at baseline, i.e. only in FI_49_) | < 5 medicines = 0, ≥ 5 medicines = 1 | 1 = 15.7 | - |
| Body mass index (BMI) deficit (measured only at baseline, i.e. only in FI_49_) | BMI < 18.5 or BMI > 30 = 1, BMI ≥ 18.5 & BMI ≤ 30 = 0 | 1 = 25.7 | 1.2 |
| Bedrest | no = 0, yes = 1 | 1 = 8.9 | - |
| Dizziness | no = 0, yes = 1 | 1 = 20.7 | - |
| Fall(s) | no = 0, yes = 1 | 1 = 5.2 | - |
| Pain | Pain rating from 0-10. 0 = 0, ≥ 1 & ≤ 3 = 0.5, ≥ 4 = 1 | 0 = 26.1  0.5 = 35.2  1 = 38.7 | - |
| Tiredness | Never = 0,  sometimes = 0.5,  always/often = 1 | 0 = 43.0  0.5 = 42.0  1 = 15.0 | - |
| Vision | Excellent = 0, very good = 0.25, good = 0.50, moderate = 0.75, poor = 1 | 0 = 10.4  0.25 = 38.0  0.50 = 35.1  0.75 = 13.4  1 = 3.1 | 0.5 |
| Hearing | Excellent = 0, very good = 0.25, good = 0.50, moderate = 0.75, poor = 1 | 0 = 12.7  0.25 = 36.2  0.50 = 30.8  0.75 = 16.9  1 = 3.3 | 0.2 |
| Attention | 10 words immediate recall test.  ≥ 5 = 0, < 5 = 1 | 1 = 20.0 | - |
| Memory | 10 word delayed recall test.  ≥ 4 = 0, < 4 = 1 | 1 = 32.6 | - |
| Physical inactivity | Moderate physical activity: “Every day/almost every day” and “multiple times a week” = 0, “once per week” & “less often” = 1 | 1 = 21.4 | - |
| Doctor told you had: Heart problem (myocardial infarction, coronary thrombosis, other problem including congestive heart failure) | no = 0, yes = 1 | 1 = 15.3 | - |
| Doctor told you had: High blood pressure or hypertension | no = 0, yes = 1 | 1 = 48.6 | - |
| Doctor told you had: Stroke or cerebral vascular disease | no = 0, yes = 1 | 1 = 4.7 | - |
| Doctor told you had: Diabetes or high blood sugar | no = 0, yes = 1 | 1 = 19.5 | - |
| Doctor told you had: Chronic lung disease such as chronic bronchitis or emphysema | no = 0, yes = 1 | 1 = 9.9 | - |
| Doctor told you had: Cancer or malignant tumour, including leukaemia or lymphoma | no = 0, yes = 1 | 1 = 5.6 | - |
| Doctor told you had: Arthritis, including osteoarthritis, or rheumatism | no = 0, yes = 1 | 1 = 27.0 | - |
| Doctor told you had: Chronic renal disease | no = 0, yes = 1 | 1 = 2.8 | - |
| Doctor told you had: Alzheimer's disease, dementia or any other serious memory impairment | no = 0, yes = 1 | 1 = 3.1 | - |
| Doctor told you had: Mental health problem, e.g. depression, nervous problems, anxiety etc. | no = 0, yes = 1 | 1 = 7.3 | - |
| Difficulty dressing | no = 0, yes = 1 | 1 = 12.2 | - |
| Difficulty walking across room | no = 0, yes = 1 | 1 = 8.0 | 0.5 |
| Difficulty bathing/showering | no = 0, yes = 1 | 1 = 9.9 | - |
| Difficulty eating | no = 0, yes = 1 | 1 = 3.8 | - |
| Difficulty going in/out of bed | no = 0, yes = 1 | 1 = 7.5 | - |
| Difficulty using toilet | no = 0, yes = 1 | 1 = 3.5 | - |
| Difficulty preparing a warm meal | no = 0, yes = 1 | 1 = 5.4 | - |
| Difficulty shopping groceries | no = 0, yes = 1 | 1 = 11.8 | 0.5 |
| Difficulty using telephone | no = 0, yes = 1 | 1 = 1.6 | 0.7 |
| Difficulty taking medicine | no = 0, yes = 1 | 1 = 1.9 | 1.6 |
| Difficulty walking 100 meters | no = 0, yes = 1 | 1 = 12.5 | 0.7 |
| Difficulty taking one flight of stairs | no = 0, yes = 1 | 1 = 23.6 | 0.7 |
| Difficulty reaching or extending your arms above shoulder level | no = 0, yes = 1 | 1 = 14.8 | - |
| Difficulty lifting or carrying weights over 10 pounds/5 kilos, like a heavy  bag of groceries | no = 0, yes = 1 | 1 = 27.8 | 0.5 |
| Lonely | never/rarely = 0  sometimes = 0.5  often/always = 1 | 0 = 76.8  0.5 = 17.6  1 = 5.6 | - |
| Difficulty concentrating | never/rarely = 0  sometimes = 0.5  often/always = 1 | 0 = 71.4  0.5 = 25.8  1 = 2.8 | - |
| Depressed | never/rarely = 0  sometimes = 0.5  often/always = 1 | 0 = 69.0  0.5 = 26.1  1 = 4.9 | - |
| Everything takes effort | never/rarely = 0  sometimes = 0.5  often/always = 1 | 0 = 66.9  0.5 = 23.9  1 = 9.2 | - |
| Sleep problems | never/rarely = 0  sometimes = 0.5  often/always = 1 | 0 = 52.7  0.5 = 35.3  1 = 12.0 | 0.2 |
| Could not get going | never/rarely = 0  sometimes = 0.5  often/always = 1 | 0 = 65.7  0.5 = 27.7  1 = 6.6 | - |
| Sad | never/rarely = 0  sometimes = 0.5  often/always = 1 | 0 = 72.3  0.5 = 23.0  1 = 4.7 | - |
| Poor appetite | never/rarely = 0  sometimes = 0.5  often/always = 1 | 0 = 89.0  0.5 = 8.0  1 = 3.1 | - |
| Numeracy problem (serial 7s) | wrong = 1  correct = 0 | 0 = 83.6  1 = 16.4 | - |
| Weak grip strength (measured only at baseline, i.e. only in FI_49_) | Grip strength (GS) = maximum grip strength in kg over four trials (2 left, 2 right).  Men:  GS ≤ 29 & BMI ≤ 24 = 1  GS ≤ 30 & BMI >24 & BMI ≤ 28 = 1  GS ≤ 32 & BMI > 28 = 1  Women:  GS ≤ 17 & BMI ≤ 23 = 1  GS ≤ 17.3 & BMI >23 & BMI ≤ 26 = 1  GS ≤ 18 & BMI > 26 & BMI ≤ 29 = 1  GS ≤ 21 & BMI > 29 = 1  Participants who cannot perform the test = 1 | 1 = 31.0 | 1.4 |
| Slow gait speed (measured only at baseline, i.e. only in FI_49_) | Gait speed in seconds = maximum of two trials over 2.5 meters. Lowest 20 % = 1- Participants who cannot perform the test = 1 | 1 = 18.4 | 1.6 |
| Slow chair rises (measured only at baseline, i.e. only in FI_49_) | Time in seconds for 5 chair rises. Age ≤ 79 & time > 14 = 1, age ≥ 80 & time > 16 = 1. Participants who cannot perform the test = 1 | 1 = 34.4 | 1.2 |

In physical performance tests, participants who stated that they are unable to participate were counted as having the respective deficit.

Supplementary Table 2: Criteria distinguishing reflective and formative concepts^1–3^ and their application to the FI

| Criterion | Application to frailty index |
| --- | --- |
| Nature of construct: reflective = latent construct exists independently of measurement; formative = latent constructs exists only in/through measurement | Older adults’ frailty level reflects a biological/medical reality that exists independently of the tools applied to measure frailty 🡺 more likely **reflective** |
| Direction of causality between construct and indicators: reflective = underlying construct causes indicators; formative = indicators cause/constitute construct | The underlying level of frailty, that is, the age-related damages and declines in repair ranging from cellular-, tissue-, and organ- to system-level^4,5^ cause the clinically visible health deficits of the FI. In other words, the measured health deficits are manifestations rather than defining characteristics of frailty 🡺 more likely **reflective** |
| Interchangeability of indicators: reflective = indicators are exchangeable; formative = indicators are not exchangeable and change would alter meaning of construct | Indicators of the FI are interchangeable^6^ in principle and dropping one indicator does not alter the conceptual domain of the FI^6,7^, although multiple physiological systems and multiple domains should be covered 🡺 more likely **reflective** |
| Covariation among indicators: reflective = indicators are all positively inter-correlated, formative = inter-correlation can have any pattern | Health deficits of the FI are considered to be “predominantly interdependent”^8,9^, i.e. each of them is linked to many others, although a change in one indicator is not expected to result in changes in all other indicators 🡺 more likely **reflective** |
| Shared antecedents and consequences of indicators: reflective = yes, formative = no | All health deficits of the FI share a common theme as they are all age-related health damages, which together reflect biological aging^9,10^. As for common consequences, negative health-related outcomes have been shown to be consistently associated with the FI, independently of which health deficits are selected^7^. However, not all health deficits will be predictive on their own 🡺 more likely **reflective** |

Supplementary Methods 2: Calculation of internal consistency

Assessment of internal consistency started with calculating the matrix of polychoric correlation coefficients^11^, which assumes that the categorical health deficits have an underlying continuous and normally-distributed character. This assumption likely holds for many of the health deficits, except the diagnosed chronic diseases. Based on the polychoric correlation matrix, we calculated the mean overall correlation among all health deficits as well as health deficit specific correlations. As a quality criterion, Clark & Watson^12^ suggest an average inter-item correlation between 0.15-0.50, although they emphasize that for broad higher order constructs, a lower mean correlation (0.15-0.20) is desirable. Next, we assessed the correlations between single health deficits and the FI using polyserial correlation coefficients^13^. As a quality criterion, Streiner et al.^14(p93)^ suggested a range between 0.20-0.80 for these correlations.

Internal consistency is most often assessed with Cronbach’s coefficient alpha^15^. Coefficient alpha, however, makes multiple assumptions, chief among them, that all indicators are equally strong related with the overall construct^16^. Coefficient omega^17^ has been suggested as a more robust alternative^18^ that is compatible with differential indicator loadings. Both coefficients, however, require that all indicators load on the same underlying construct, i.e. that the overall scale is unidimensional. We used a model-based approach^16^, i.e. confirmatory factor analysis (CFA) on the health deficits of FI_49_ to evaluate unidimensionality. Given the multi-domain nature of the FI as it combines information on physical, cognitive, and mental health from a number of different instruments^31^, we tested whether the FI was best represented by a unidimensional 1-factor CFA model, a multidimensional first-order model of three (physical, mental, cognitive) separated but correlating domains, or a bifactor model, which posits that all health deficits load on a general factor (of frailty) as well as group factors (physical, mental cognitive) that capture remaining domain-specific variance. Model comparison was based on multiple global fit indices: Scaled Chi² test, the Tucker Lewis Index (TLI), the Comparative Fit Index (CFI), the Root Mean Square Error of Approximation (RMSEA), and the Standardized Root Mean Square Residual (SRMR). We also compared the loadings of the health deficits between the unidimensional and the bifactor model to ascertain the level of multidimensionality. A high correlation between the loadings of the unidimensional model and the general factor of the bifactor model would indicate that the level of multidimensionality is negligible, and unidimensionality can be assumed for practical purposes^19,20^. In the latter case, we would be able to meaningfully interpret the internal consistency estimate and use the single-score instrument of the FI. For CFA, all health deficits were treated as categorical, either dichotomous (0/1) or ordinal (e.g. 0, 0.25, 0.50, 0.75, 1), and estimation was done with a diagonally weighted least squares procedure (WLSMV) with robust standard errors using R-package lavaan (0.6-7)^21^. Internal consistency – which is expressed as a dimensionless number ranging between 0 (completely unreliable) and 1 (perfectly reliable) – was calculated using R-package semTools (0.5-6).

Supplementary Methods 3: Calculation of test-retest reliability and measurement error

We assessed both reliability and measurement error^14^ based on the longitudinal FI_44_ assessments. For reliability, we first calculated and plotted Pearson correlation coefficients between adjacent waves. However, since correlation coefficients may conceal systematic differences^23^ between repeated FI_44_ measurements, we focussed on the intraclass correlation coefficient (ICC)^24,25^. Specifically, we used a two-way random effects, single measurement, and absolute agreement model (ICC2(A,1)). Since there was missing data across the seven repeated FI_44_ measurements and since repeated assessments were not conducted exactly at the same intervals, we used a linear mixed regression model rather than the more restrictive ANOVA-based approach to estimate the variances required for the calculation of the ICC. The corresponding mixed regression model was formulated as

$${FI}_{ij}=\gamma_{00}+u_{i} {+ u}_{j}+\varepsilon_{ij}$$

where the frailty index value of an individual *i* at time-point *j* is a function of the overall mean FI level ($\gamma_{00}$), the variability across individuals ($u_{i}$) as well as across time$(u_{j}$), and the residual error term ($\varepsilon_{ij}$). This model was estimated with R-package lme4 (v1.1-27.1)^26^. The ICC was then calculated^27,28^ as the proportion of between-person FI variance ($\sigma_{i}^{2})$compared to the overall FI variance:

$$ICC= \frac{\sigma_{i}^{2}}{\sigma_{i}^{2}+ \sigma_{j}^{2}+ \sigma_{residual}^{2}}$$

95% confidence intervals for the ICC were estimated with a bootstrap procedure. Just like the measures of internal consistency, the ICC can range between 0-1.

Measurement error, i.e. the standard error of measurement (SEM)^27^ was calculated as

$$SEM= \sqrt{\sigma_{j}^{2}+}\sigma_{residuals}^{2}$$

and 95% confidence intervals were again bootstrapped. In contrast to the ICC, the SEM is expressed in the same metric as the underlying construct. Furthermore, we created Bland-Altman plots^29^ based on R package blandr (0.5-3)^30^ to visualize both systematic bias between adjacent FI assessments and calculated the limits of agreement (LOA) which include 95% of the differences between paired measurements. Finally, based on the SEM and its 95% confidence interval, we calculated the smallest detectable change (SDC), i.e. the smallest change needed to ensure that a change in the FI of an older person can be considered as “real” and not just measurement error^28^, as

$$SDC=SEM*1.96* \sqrt{2}$$

Supplementary Figure 2: Descriptive statistics of the longitudinal frailty index (FI_44_) by interview mode


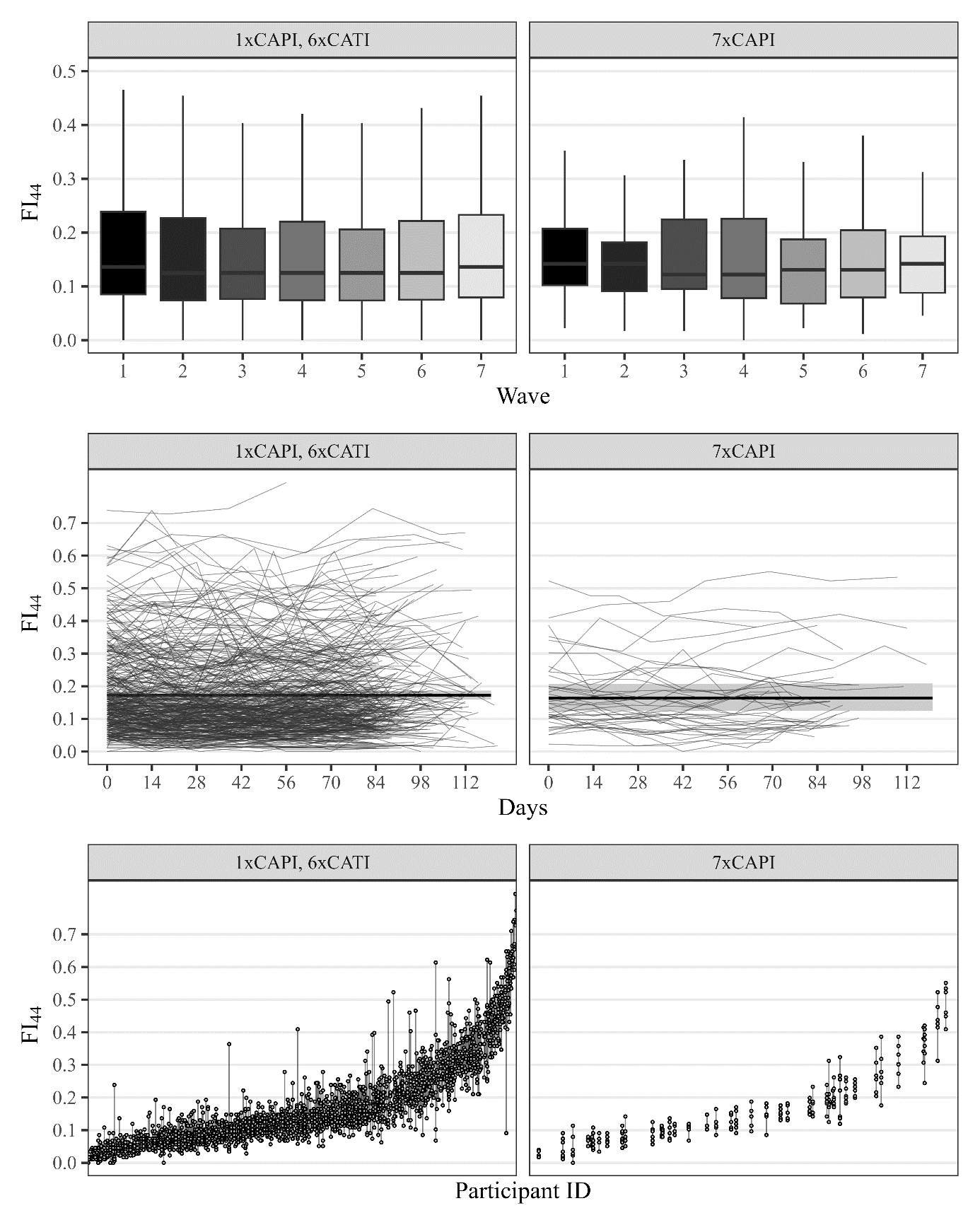


CAPI = computer-assisted personal interview, CATI = computer-assisted telephone interview. n=386 for 1xCAPI, 6xCATI, and n=40 for 7xCAPI

Supplementary Table 3: Model fit comparison (confirmatory factor analysis)

|  | Fit indices |  |  |  |  |
| --- | --- | --- | --- | --- | --- |
| Model | Scaled Chi² | RMSEA | CFI | TLI | SRMR |
| 1-factor model | Chi²=1,621.98  df=1,124  p-value<0.001 | 0.034 | 0.923 | 0.919 | 0.126 |
| First order model | Chi²=1,409,63  df = 1,121  p-value<0.001 | 0.026 | 0.955 | 0.953 | 0.114 |
| Bifactor model | Chi²=1,278,96  df=1,075  p-value<0.001 | 0.022 | 0.968 | 0.965 | 0.107 |

The scaled Chi² difference test found the bifactor model to provide a significantly better model (Chi² difference = 150, degrees of freedom differences = 46, p<0.001) than the second best model, the first order model, which again fitted better than the single-factor model (Chi² difference = 120, degrees of freedom differences = 3, p<0.001). df = degrees of freedom, RMSEA = Root Mean Square Error of Approximation, CFI = Comparative Fit Index, TLI = Tucker Lewis Index, SRMR = Standardizes Root Mean Square Residual

Supplementary Table 4: Bivariate polyserial correlations and factor loadings between frailty index (FI_49_) and specific health deficits

| Health deficit | Bivariate correlation | Factor loadings* |
| --- | --- | --- |
| Self-rated health | 0.73 | 0.83 |
| Polypharmacy | 0.62 | 0.84 |
| BMI deficit | 0.33 | 0.31 |
| Bedrest | 0.52 | 0.69 |
| Dizziness | 0.53 | 0.53 |
| Fall(s) | 0.30 | 0.35 |
| Pain | 0.60 | 0.70 |
| Tiredness | 0.66 | 0.64 |
| Vision | 0.31 | 0.28 |
| Hearing | 0.26 | 0.19 |
| Attention | 0.47 | 0.47 |
| Memory | 0.44 | 0.41 |
| Physical inactivity | 0.55 | 0.62 |
| Heart problem (myocardial infarction, coronary thrombosis, other heart problems) | 0.34 | 0.37 |
| High blood pressure or hypertension | 0.31 | 0.27 |
| Stroke or cerebral vascular disease | 0.28 | 0.28 |
| Diabetes or high blood sugar | 0.30 | 0.27 |
| Chronic lung disease such as chronic bronchitis or emphysema | 0.32 | 0.34 |
| Cancer or malignant tumour, including leukaemia or lymphoma | 0.19 | 0.14 |
| Arthritis, including osteoarthritis, or rheumatism | 0.41 | 0.44 |
| Chronic renal disease | 0.26 | 0.32 |
| Alzheimer's disease, dementia or any other serious memory impairment | 0.40 | 0.47 |
| Mental health problem, e.g. depression, nervous problems, anxiety etc. | 0.39 | 0.36 |
| Difficulty dressing | 0.59 | 0.70 |
| Difficulty walking across room | 0.67 | 0.81 |
| Difficulty Bathing/showering | 0.67 | 0.82 |
| Difficulty eating | 0.40 | 0.57 |
| Difficulty going in/out of bed | 0.57 | 0.83 |
| Difficulty using toilet | 0.59 | 0.88 |
| Difficulty preparing a warm meal | 0.67 | 0.87 |
| Difficulty shopping groceries | 0.73 | 0.84 |
| Difficulty using telephone | 0.34 | 0.56 |
| Difficulty taking medicine | 0.54 | 0.92 |
| Difficulty walking 100 meters | 0.67 | 0.71 |
| Difficulty taking one flight of stairs | 0.69 | 0.74 |
| Difficulty reaching or extending your arms above shoulder level | 0.59 | 0.66 |
| Difficulty lifting or carrying weights over 10 pounds/5 kilos | 0.70 | 0.75 |
| Lonely | 0.43 | 0.38 |
| Difficulty concentrating | 0.43 | 0.42 |
| Depressed | 0.52 | 0.48 |
| Everything takes effort | 0.66 | 0.73 |
| Sleep problems | 0.37 | 0.32 |
| Could not get going | 0.65 | 0.67 |
| Sad | 0.45 | 0.44 |
| Poor appetite | 0.44 | 0.54 |
| Numeracy problem | 0.33 | 0.35 |
| Weak grip strength | 0.57 | 0.55 |
| Slow gait speed | 0.63 | 0.74 |
| Slow chair rises | 0.66 | 0.70 |

* Factor loadings refer to the loadings on general factor in the bifactor model.

Supplementary Figure 3: Polychoric correlation matrix of health deficits in baseline frailty index (FI_49_)


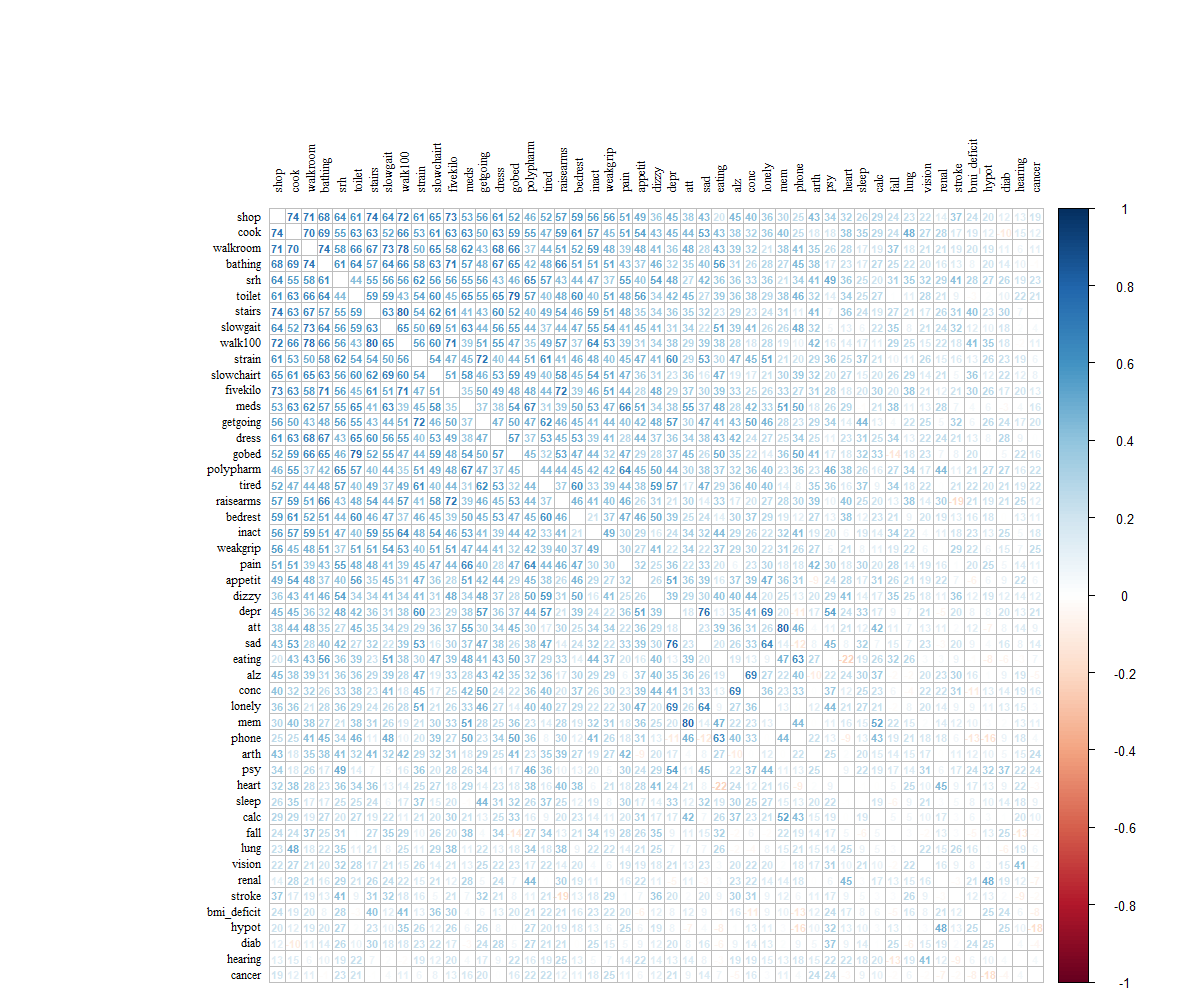


Numbers refer to polychoric correlation coefficients multiplied with 100 for easier display.

Supplementary references

1. Jarvis CB, MacKenzie SB, Podsakoff PM. A Critical Review of Construct Indicators and Measurement Model Misspecification in Marketing and Consumer Research. *Journal of Consumer Research*. 2003;30(2):199-218. doi:10.1086/376806

2. Coltman T, Devinney TM, Midgley DF, Venaik S. Formative versus reflective measurement models: Two applications of formative measurement. *Journal of Business Research*. 2008;61:1250-1262. doi:10.1016/j.jbusres.2008.01.013

3. Fleuren BPI, van Amelsvoort LGPM, Zijlstra FRH, de Grip A, Kant Ij. Handling the reflective-formative measurement conundrum: a practical illustration based on sustainable employability. *Journal of Clinical Epidemiology*. 2018;103:71-81. doi:10.1016/j.jclinepi.2018.07.007

4. Howlett SE, Rutenberg AD, Rockwood K. The degree of frailty as a translational measure of health in aging. *Nat Aging*. 2021;1(8):651-665. doi:10.1038/s43587-021-00099-3

5. Rockwood K, Mitnitski A, Howlett SE. Frailty: Scaling from Cellular Deficit Accumulation? *Interdiscip Top Gerontol Geriatr*. 2015;41:1-14. doi:10.1159/000381127

6. Searle SD, Mitnitski A, Gahbauer EA, Gill TM, Rockwood K. A standard procedure for creating a frailty index. *BMC Geriatrics*. 2008;8(1):24. doi:10.1186/1471-2318-8-24

7. Rockwood K, Mitnitski A, Song X, Steen B, Skoog I. Long-term risks of death and institutionalization of elderly people in relation to deficit accumulation at age 70. *J Am Geriatr Soc*. 2006;54(6):975-979. doi:10.1111/j.1532-5415.2006.00738.x

8. Mitnitski A, Song X, Rockwood K. Assessing biological aging: the origin of deficit accumulation. *Biogerontology*. 2013;14(6):709-717. doi:10.1007/s10522-013-9446-3

9. Mitnitski A, Rockwood K. Aging as a Process of Deficit Accumulation: Its Utility and Origin. *Aging and Health - A Systems Biology Perspective*. 2015;40:85-98. doi:10.1159/000364933

10. Mitnitski AB, Graham JE, Mogilner AJ, Rockwood K. Frailty, fitness and late-life mortality in relation to chronological and biological age. *BMC Geriatr*. 2002;2:1. doi:10.1186/1471-2318-2-1

11. Drasgow F. Polychoric and Polyserial Correlations. In: *Encyclopedia of Statistical Sciences*. John Wiley & Sons, Ltd; 2004. doi:10.1002/0471667196.ess2014

12. Clark LA, Watson D. Constructing validity: Basic issues in objective scale development. *Psychological Assessment*. 1995;7:309-319. doi:10.1037/1040-3590.7.3.309

13. Olsson U, Drasgow F, Dorans NJ. The polyserial correlation coefficient. *Psychometrika*. 1982;47(3):337-347. doi:10.1007/BF02294164

14. Streiner DL, Norman GR, Cairney J. *Health Measurement Scales: A Practical Guide to Their Development and Use, 5th Ed*. Oxford University Press; 2015:xiii, 399. doi:10.1093/med/9780199685219.001.0001

15. Cronbach LJ. Coefficient alpha and the internal structure of tests. *Psychometrika*. 1951;16(3):297-334. doi:10.1007/BF02310555

16. Revelle W, Condon DM. Reliability from α to ω: A tutorial. *Psychological Assessment*. 2019;31:1395-1411. doi:10.1037/pas0000754

17. McDonald RP. *Test Theory: A Unified Treatment*. Lawrence Erlbaum Associates Publishers; 1999:xi, 485.

18. McNeish D. Thanks coefficient alpha, we’ll take it from here. *Psychol Methods*. 2018;23(3):412-433. doi:10.1037/met0000144

19. Reise SP, Morizot J, Hays RD. The role of the bifactor model in resolving dimensionality issues in health outcomes measures. *Qual Life Res*. 2007;16 Suppl 1:19-31. doi:10.1007/s11136-007-9183-7

20. Reise SP, Bonifay WE, Haviland MG. Scoring and modeling psychological measures in the presence of multidimensionality. *J Pers Assess*. 2013;95(2):129-140. doi:10.1080/00223891.2012.725437

21. Rosseel Y. lavaan: An R Package for Structural Equation Modeling. *Journal of Statistical Software*. 2012;48:1-36. doi:10.18637/jss.v048.i02

22. Kelley K. Confidence Intervals for Standardized Effect Sizes: Theory, Application, and Implementation. *Journal of Statistical Software*. 2007;20:1-24. doi:10.18637/jss.v020.i08

23. Aldridge VK, Dovey TM, Wade A. Assessing test-retest reliability of psychological measures: Persistent methodological problems. *European Psychologist*. 2017;22:207-218. doi:10.1027/1016-9040/a000298

24. McGraw KO, Wong SP. Forming inferences about some intraclass correlation coefficients. *Psychological Methods*. 1996;1:30-46. doi:10.1037/1082-989X.1.1.30

25. Koo TK, Li MY. A Guideline of Selecting and Reporting Intraclass Correlation Coefficients for Reliability Research. *J Chiropr Med*. 2016;15(2):155-163. doi:10.1016/j.jcm.2016.02.012

26. Bates D, Mächler M, Bolker B, Walker S. Fitting Linear Mixed-Effects Models Using lme4. *Journal of Statistical Software*. 2015;67:1-48. doi:10.18637/jss.v067.i01

27. de Vet HCW, Terwee CB, Knol DL, Bouter LM. When to use agreement versus reliability measures. *J Clin Epidemiol*. 2006;59(10):1033-1039. doi:10.1016/j.jclinepi.2005.10.015

28. Weir JP. Quantifying test-retest reliability using the intraclass correlation coefficient and the SEM. *J Strength Cond Res*. 2005;19(1):231-240. doi:10.1519/15184.1

29. Altman DG, Bland JM. Measurement in Medicine: The Analysis of Method Comparison Studies. *Journal of the Royal Statistical Society Series D (The Statistician)*. 1983;32(3):307-317. doi:10.2307/2987937

30. Datta D. blandr: a Bland-Altman Method Comparison package for R. Published online 2017. https://github.com/deepankardatta/blandr

31. Hoogendijk EO, Theou O, Rockwood K, Onwuteaka-Philipsen BD, Deeg DJH, Huisman M. Development and validation of a frailty index in the Longitudinal Aging Study Amsterdam. *Aging Clin Exp Res*. 2017;29(5):927-933. doi:10.1007/s40520-016-0689-0
